# Safety and efficacy of vinorelbine in combination with pertuzumab and trastuzumab for first-line treatment of patients with HER2-positive locally advanced or metastatic breast cancer: VELVET Cohort 1 final results

**DOI:** 10.1186/s13058-016-0773-6

**Published:** 2016-12-13

**Authors:** Edith A. Perez, José Manuel López-Vega, Thierry Petit, Claudio Zamagni, Valerie Easton, Julia Kamber, Eleonora Restuccia, Michael Andersson

**Affiliations:** 1 US Medical Affairs, Genentech, Inc., South San Francisco, CA 94080 USA; 2 Hospital Universitario Marqués de Valdecilla, Santander, Spain; 3 Department of Medical Oncology, Paul Strauss Cancer Center and University of Strasbourg, Strasbourg, France; 4 Policlinico S. Orsola-Malpighi Hospital, Bologna, Italy; 5 F. Hoffmann-La Roche Ltd., Basel, Switzerland; 6 Department of Oncology, Rigshospitalet, Copenhagen, Denmark

**Keywords:** Vinorelbine, Trastuzumab, Pertuzumab, Locally advanced breast cancer, Metastatic breast cancer

## Abstract

**Background:**

Pertuzumab, trastuzumab, and docetaxel is standard of care for first-line treatment of HER2-positive metastatic breast cancer (MBC). However, alternative chemotherapy partners are required to align with patient/physician preferences and to increase treatment flexibility. We report VELVET Cohort 1 results in which the efficacy and safety of pertuzumab and trastuzumab, administered sequentially in separate infusions, followed by vinorelbine, were evaluated. Cohort 2, where pertuzumab and trastuzumab were administered in a single infusion, followed by vinorelbine, recruited after Cohort 1 was fully enrolled, will be reported later.

**Methods:**

In this multicenter, two-cohort, open-label, phase II study, patients with HER2-positive locally advanced or MBC who had not received chemotherapy or biological therapy for their advanced disease received 3-weekly pertuzumab (840 mg loading, 420 mg maintenance doses) and trastuzumab (8 mg/kg loading, 6 mg/kg maintenance doses), followed by vinorelbine (25 mg/m^2^ initial dose, 30–35 mg/m^2^ maintenance doses) on days 1 and 8 or 2 and 9 of each 3-weekly cycle. Study treatment was given until investigator-assessed disease progression or unacceptable toxicity. The primary endpoint was investigator-assessed objective response rate (ORR) in patients with measurable disease at baseline per RECIST v1.1. Secondary endpoints included progression-free survival (PFS) and safety.

**Results:**

Cohort 1 enrolled 106 patients. Investigator-assessed ORR was 74.2% (95% CI 63.8–82.9) in intent-to-treat patients with measurable disease (89/106 [84.0%]). Median PFS was 14.3 months (95% CI 11.2–17.5) in the intent-to-treat population. Treatment was reasonably well tolerated, with no unexpected toxicities. Diarrhea (61/106 patients [57.5%]) and neutropenia (54/106 [50.9%]) were the most common adverse events (AEs); neutropenia (33/106 [31.1%]) and leukopenia (14/106 [13.2%]) were the most common grade ≥3 AEs. Serious AEs were reported in 32/106 (30.2%) patients. AEs led to study drug discontinuation in 36/106 patients (34.0%). Eighteen of 106 patients (17.0%) had AEs suggestive of congestive heart failure; however, there were no confirmed cases.

**Conclusions:**

The vinorelbine, pertuzumab, and trastuzumab combination is active and reasonably well tolerated. This regimen offers an alternative for patients who cannot receive docetaxel for first-line treatment of HER2-positive locally advanced or MBC.

**Trial registration:**

ClinicalTrials.gov: NCT01565083, registered on 26 March 2012.

**Electronic supplementary material:**

The online version of this article (doi:10.1186/s13058-016-0773-6) contains supplementary material, which is available to authorized users.

## Background

HER2 overexpression/amplification occurs in 15–20% of breast cancers [1] and is associated with poor prognosis [2]. Trastuzumab and pertuzumab are humanized monoclonal antibodies that bind HER2 subdomains IV and II, respectively [3, 4]. Treatment with both agents offers more comprehensive signaling blockade than either agent alone, due to distinct but complementary modes of action [5, 6].

In the pivotal CLEOPATRA trial, first-line treatment with pertuzumab in combination with trastuzumab and docetaxel significantly increased independently assessed progression-free survival (PFS) by 6.1 months (hazard ratio 0.62, *P* < 0.001) [7] and overall survival (OS) by 15.7 months (hazard ratio 0.68, *P* < 0.001) [8], compared with trastuzumab and docetaxel in patients with HER2-positive metastatic breast cancer. Docetaxel is an established and effective cytotoxic agent but for some selected cases physicians and patients may prefer a different chemotherapy partner due to prior docetaxel treatment, or because of its toxicity profile, which may make it unsuitable for some patients [9, 10]. Therefore, to increase flexibility in treatment decision-making, there is a need to investigate other chemotherapies as alternative partners for pertuzumab and trastuzumab.

The vinca alkaloid vinorelbine demonstrates synergistic activity with trastuzumab against HER2-overexpressing breast cancer cells [11]. Multiple trials have shown that first-line treatment with vinorelbine plus trastuzumab for HER2-positive metastatic breast cancer is efficacious and well tolerated [12–22]. Furthermore, the HERNATA study demonstrated that docetaxel and trastuzumab were not superior to vinorelbine and trastuzumab in terms of efficacy in patients with HER2-positive advanced breast cancer, but patients who received vinorelbine experienced fewer grade 3 and 4 adverse events (AEs) than those treated with docetaxel [22]. Vinorelbine may offer an alternative to docetaxel but has not yet been tested in combination with dual HER2-targeted therapy.

The VELVET study is evaluating the efficacy and safety of pertuzumab, trastuzumab, and vinorelbine for the first-line treatment of HER2-positive locally advanced or metastatic breast cancer. Trastuzumab and pertuzumab were administered either as separate intravenous infusions (Cohort 1) or co-administered in a single infusion bag (Cohort 2), followed by vinorelbine. The two-cohort design was chosen so that in addition to evaluating the efficacy of vinorelbine as a chemotherapy partner for pertuzumab and trastuzumab, the feasibility of their co-administration, which has the potential to increase convenience and minimize clinic time for patients, could also be assessed for the first time. Here we report the final results for Cohort 1.

## Methods

### Study design

**Fig. 1:**
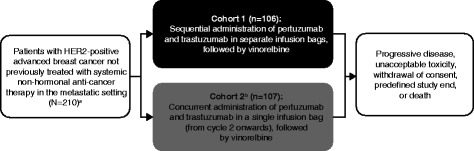
Study design. ^a^ Sample size was based on assuming a best overall response of 70–80% in each cohort. ^b^ Recruitment into Cohort 2 began after Cohort 1 had finished enrolling patients

VELVET is a two-cohort, open-label, multicenter phase II, proof-of-concept trial involving patients with HER2-positive locally advanced or metastatic breast cancer who had not received chemotherapy or biological therapy for advanced disease. Patients in Cohort 1 received pertuzumab (PERJETA®; F. Hoffmann-La Roche Ltd., Basel, Switzerland), trastuzumab (Herceptin®; F. Hoffmann-La Roche Ltd.), and vinorelbine (Bendarelbin; Bendalis GmbH, Oberhaching, Germany) as separate infusion bags; patients in Cohort 2 received pertuzumab and trastuzumab as a single infusion bag (from cycle 2 onwards), followed by vinorelbine (Fig. 1). Cohort 2 will be analyzed separately and will be reported elsewhere. Safety reviews were performed by an independent data monitoring committee (IDMC) in May 2012 after the first patient was enrolled into Cohort 1, in January 2013 when all patients had been enrolled into Cohort 1, and in April 2013 when 102 patients had received at least two cycles of study treatment. After this, recruitment into Cohort 2 began and the IDMC performed reviews of accumulating safety and efficacy data every 6 months. The final review was in March 2015.

### Patients

Eligible patients were ≥18 years, had HER2-positive locally advanced (not amenable to curative resection) or metastatic breast cancer as assessed by local laboratory on primary or metastatic tumor by immunohistochemistry (with 3+ indicating positivity) or in situ hybridization (with a HER2/chromosome 17 ratio of ≥2.0 indicating positivity), and measurable and/or non-measurable disease, evaluable according to Response Evaluation Criteria In Solid Tumors (RECIST) version 1.1 [23]. HER2 positivity was subsequently analyzed centrally (Targos Molecular Pathology GmbH, Kassel, Germany) but central results were not required prior to enrollment. Additional inclusion criteria were Eastern Cooperative Oncology Group (ECOG) performance status 0 or 1, left ventricular ejection fraction (LVEF) of ≥55% at baseline, and a life expectancy of ≥12 weeks. Patients may have received ≤2 lines of hormonal therapy for metastatic or locally recurrent disease, one of which could have been in combination with everolimus.

Exclusion criteria included prior systemic non-hormonal therapy in the metastatic or locally advanced setting, prior treatment with anti-HER2 agents, except with neoadjuvant or adjuvant trastuzumab and/or lapatinib, disease progression while receiving neoadjuvant or adjuvant trastuzumab and/or lapatinib, a disease-free interval of <6 months from completion of neoadjuvant or adjuvant non-hormonal therapy to time of disease recurrence, uncontrolled central nervous system metastases, uncontrolled hypertension, and clinically significant cardiovascular disease.

### Procedures

Patients received a pertuzumab loading dose of 840 mg on day 1 of cycle 1, followed by 420 mg on day 1 of subsequent 3-weekly cycles. Trastuzumab was administered at a loading dose of 8 mg/kg on day 2 of cycle 1, followed by 6 mg/kg on day 1 or 2 of subsequent 3-weekly cycles. Vinorelbine was administered at an initial dose of 25 mg/m^2^ on days 2 and 9 of cycle 1, followed by 30–35 mg/m^2^ on days 1 and 8 or days 2 and 9 of subsequent 3-weekly cycles. All were administered intravenously and were given until investigator-assessed disease progression or unacceptable toxicity. In an effort to appropriately monitor safety during the initial infusion (cycle 1), pertuzumab had to be administered on day 1 and trastuzumab followed by vinorelbine on day 2. Vinorelbine was administered in line with product labeling. The dosing schedule for vinorelbine was the same as that used in HERNATA [22], with the exception of cycle 1, in which vinorelbine was administered at a lower dose to evaluate its tolerability with the addition of pertuzumab to the treatment regimen. For vinorelbine, in the case of grade 3 to 4 toxicities, treatment was delayed until toxicity improved to grade 1, and then the dose was reduced to 80%. In the case of elevation of bilirubin more than two times the upper limit of normal or transaminases more than three times the upper limit of normal the dose was reduced to 50% [22]. If vinorelbine was discontinued due to toxicity, antibody therapy was continued until disease progression; if antibody therapy was discontinued due to toxicity, vinorelbine was continued until disease progression. Dose reductions were not permitted for antibody therapy.

Routine RECIST v1.1-based tumor assessments were performed at baseline, every three cycles up to 36 months, and then every six cycles until disease progression. LVEF assessments were performed at baseline and every three cycles, by echocardiogram or multi-gated acquisition scan. ECOG performance status was assessed at baseline, every three cycles, and 28 days after treatment discontinuation. Laboratory tests were performed at baseline, every cycle, and 28 days after treatment discontinuation. AEs and serious AEs (SAEs) were monitored continuously during treatment and until 28 days after treatment discontinuation, and graded according to Medical Dictionary for Regulatory Activities (MedDRA) version 18.0. Patients with SAEs that were ongoing at treatment discontinuation were followed until resolution of the event. Computed tomography or magnetic resonance imaging brain scans were carried out during screening only if brain metastases were clinically suspected and during the study if clinically indicated.

### Outcomes

The primary endpoint was investigator-assessed objective response rate (ORR) in patients with measurable disease at baseline, according to RECIST v1.1 and was based on the best overall response (BOR, the best response recorded from the start of trial treatment until disease progression/recurrence or death). Secondary endpoints included time to response, duration of response (DoR) in responders (patients with a confirmed BOR of either complete response or partial response, confirmed by two consecutive assessments), PFS, time to progression (TTP), OS, safety and tolerability, and health-related quality of life (HRQoL). HRQoL will be reported separately. Safety analyses included incidence and severity of AEs and SAEs, incidence of congestive heart failure (CHF), changes in LVEF during the study, and laboratory test abnormalities.

### Statistical analysis

Assuming a BOR rate of 70–80% per cohort, and aiming at a distance from the estimated proportion to the confidence interval (CI) limits of 8–11%, 95 patients were needed per cohort. Assuming a withdrawal rate of approximately 10%, it was planned to enroll 105 patients in each cohort (calculated using SAS software, version 9.2 and nQuery, version 6). The BOR rate of 70–80% was chosen based on published data [7]. All analyses presented are descriptive. Efficacy analyses were performed in the intent-to-treat (ITT) population (all enrolled patients). The primary endpoint, ORR, was summarized by the number and percentage of responders, together with two-sided 95% Clopper-Pearson CIs in ITT patients with measurable disease at baseline. The Kaplan–Meier approach was used to estimate median time to response, DoR, PFS, TTP, and OS. Predefined exploratory subgroup analyses were performed for ORR and PFS, stratified by prior trastuzumab treatment and by hormone receptor status. Sensitivity analyses were performed, excluding tumor assessments performed after the intake of new anticancer therapy (ORR, PFS, and TTP), and including progressive disease due to symptomatic deterioration (PFS and TTP). The median time on study and 95% CIs were estimated using the reverse Kaplan–Meier method. AEs were evaluated descriptively in the safety population (all patients who received at least one dose of any study treatment). The study end for each cohort is when all patients have been followed up for at least 2 years after the last patient was enrolled, within each respective cohort, unless they have been lost to follow-up, withdrawn consent, or died. This study is registered with ClinicalTrials.gov, number NCT01565083. A pertuzumab extension study (NCT02320435) opened in February 2015 to provide patients with continued access to pertuzumab.

## Results

### Patients

**Fig. 2:**
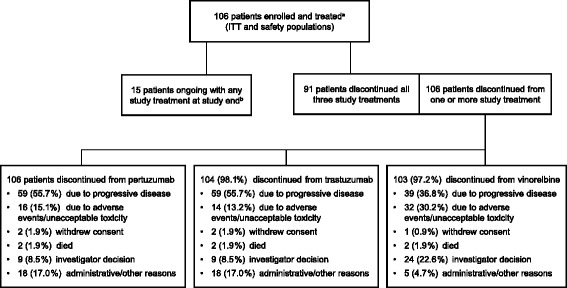
Trial profile. ^a^ In cycle 1, 106 patients received pertuzumab, 104 patients received trastuzumab, and 103 patients received vinorelbine. ^b^ The 15 patients ongoing with any study treatment at time of study closure are also counted under administrative/other reasons

From April 2012 to December 2012, 106 patients were enrolled into Cohort 1 at 44 centers in Europe and the USA and all were included in the ITT and safety populations. At data cutoff and study end for Cohort 1 (May 12, 2015), all patients had discontinued one or more study treatments; 91/106 (85.8%) patients had discontinued all three, and the remaining 15 (14.2%) patients had ongoing study treatment (Fig. 2). Progressive disease was the main reason for discontinuation. Baseline patient demographics and clinical characteristics are shown in Table 1. Almost two-thirds of patients (61.3%) had received prior systemic cancer therapy: 44/106 (41.5%) had received trastuzumab, 40/106 (37.7%) taxanes, and 41/106 (38.7%) anthracyclines. Seventy of 106 (66.0%) patients had estrogen and/or progesterone receptor-positive disease. Eighty-eight of 106 (83.0%) patients had their locally tested HER2-positive disease confirmed by the central laboratory.

**Table 1:** Baseline characteristics, intent-to-treat population

Characteristic	Cohort 1: pertuzumab, trastuzumab, and vinorelbine N = 106
Median age, years (range)	56 (30–82)
Female gender	106 (100%)
Geographical region	
Europe	91 (85.8%)
North America	15 (14.2%)
ECOG performance status	
0	74 (69.8%)
1	32 (30.2%)
Disease type at screening	
Visceral	78 (73.6%)
Non-visceral	28 (26.4%)
Disease stage at initial diagnosis	
I	12 (11.3%)
II	31 (29.2%)
III	29 (27.4%)
IV	34 (32.1%)
Disease stage at advanced breast cancer diagnosis	
Locally advanced	11 (10.4%)
Metastatic	95 (89.6%)
Hormone receptor status	
Estrogen and/or progesterone receptor-positive	70 (66.0%)
Estrogen and progesterone receptor-negative	36 (34.0%)
HER2 status, local assessment	
Immunohistochemistry	
0 or 1+	0
2+	14 (13.2%)
3+	85 (80.2%)
Not performed	7 (6.6%)
In situ hybridization	
Positive	26 (24.5%)
Negative	0
Not performed	80 (75.5%)
HER2 status, central assessment	
HER2-positive	88 (83.0%)
HER2-negative	12 (11.3%)
Not done	2 (1.9%)
Missing	4 (3.8%)
Immunohistochemistry	
0 or 1+	8 (7.5%)
2+	17 (16.0%)
3+	75 (70.8%)
Not performed	2 (1.9%)
Missing	4 (3.8%)
In situ hybridization	
Positive	80 (75.5%)
Negative	8 (7.5%)
Not performed	3 (2.8%)
Not evaluable	11 (10.4%)
Missing	4 (3.8%)
Prior systemic cancer therapy	65 (61.3%)
Taxane^a^	40 (37.7%)
Anthracycline^b^	41 (38.7%)
Trastuzumab	44 (41.5%)
Bevacizumab	1 (0.9%)

Data are number (%).

^a^Paclitaxel, docetaxel, nab-paclitaxel, or taxane (not otherwise specified)

^b^Epirubicin, doxorubicin, mitoxantrone, or anthracycline (not otherwise specified)

### Treatment exposure

Median time on study was 27.8 months (95% CI 27.2–28.6). Patients received a median of 15 pertuzumab cycles (range 1–47), 15 trastuzumab cycles (range 0–47), and 9.5 vinorelbine cycles (range 0–40). Two patients received only pertuzumab at cycle 1. The median vinorelbine dose intensity during the first six cycles was 17.95 mg/m^2^ per week (range 7.6–22.5).

### Efficacy

**Fig. 3:**
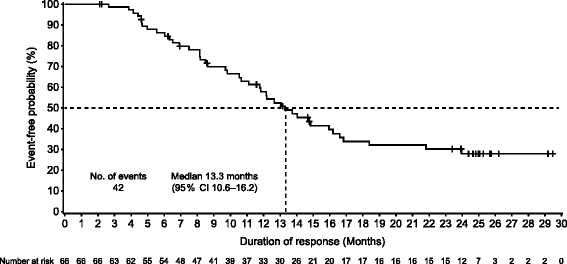
Investigator-assessed duration of response in responders, intent-to-treat population (Cohort 1). The *tick* marks indicate censoring events

**Fig. 4:**
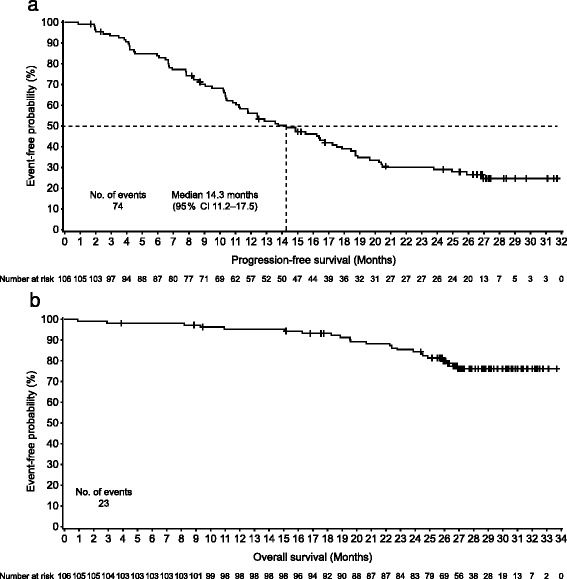
Progression-free survival (**a**) and overall survival (**b**), intent-to-treat population (Cohort 1). Median overall survival was not reached. The *tick* marks indicate censoring events

Investigator-assessed ORR in the 89/106 (84.0%) patients with measurable disease at baseline was 74.2% (95% CI 63.8–82.9, 66/89 patients); 12/89 (13.5%) patients achieved a complete response and 54/89 (60.7%) a partial response. The BOR for all patients is listed in Table 2. The median time to response was 2.1 months (range 0.0–29.0) in the 89 patients with measurable disease at baseline. Among responders (66/106 patients) the median DoR was 13.3 months (range 2.1–29.5; Fig. 3). At data cutoff, 74/106 (69.8%) patients had progressed or died and the median PFS was 14.3 months (95% CI 11.2–17.5, Table 2 and Fig. 4a). The median TTP was 14.9 months (95% CI 11.3–17.9). The median OS had not been reached by the end of the study (Fig. 4b), at which time 83/106 (78.3%) patients were still alive.

**Table 2:** Best overall response and progression-free survival for all patients, and by prior trastuzumab therapy and by hormone-receptor status, intent-to-treat population

	Cohort 1: pertuzumab, trastuzumab, and vinorelbine					
	All patients	History of prior trastuzumab therapy subgroups		Hormone receptor status subgroups		
		Prior trastuzumab therapy	No prior trastuzumab therapy	Estrogen receptor-positive and progesterone receptor-positive	Estrogen receptor-positive and progesterone receptor-negative	Estrogen receptor-negative and progesterone receptor-negative
	N = 106	n = 44	n = 62	n = 45	n = 25	n = 36
Best overall response						
Patients with measurable disease at baseline	89 (84.0%)	36 (81.8%)	53 (85.5%)	39 (86.7%)	18 (72.0%)	32 (88.9%)
Overall response rate	66 (74.2%) [63.8–82.9]	28 (77.8%) [60.8–89.9]	38 (71.7%) [57.7–83.2]	27 (69.2%) [52.4–83.0]	15 (83.3%) [58.6–96.4]	24 (75.0%) [56.6–88.5]
Complete response	12 (13.5%) [7.2–22.4]	6 (16.7%) [6.4–32.8]	6 (11.3%) [4.3–23.0]	6 (15.4%) [5.9–30.5]	1 (5.6%) [0.1–27.3]	5 (15.6%) [5.3–32.8]
Partial response	54 (60.7%) [49.7–70.9]	22 (61.1%) [43.5–76.9]	32 (60.4%) [46.0–73.5]	21 (53.8%) [37.2–69.9]	14 (77.8%) [52.4–93.6]	19 (59.4%) [40.6–76.3]
Stable disease	16 (18.0%) [10.6–27.5]	6 (16.7%) [6.4–32.8]	10 (18.9%) [9.4–32.0]	9 (23.1%) [11.1–39.3]	3 (16.7%) [3.6–41.4]	4 (12.5%) [3.5–29.0]
Progressive disease	5 (5.6%) [1.8–12.6]	2 (5.6%) [0.7–18.7]	3 (5.7%) [1.2–15.7]	2 (5.1%) [0.6–17.3]	0 [0.0–18.5]	3 (9.4%) [2.0–25.0]
Not evaluable	2 (2.2%) [0.3–7.9]	0 [0.0–9.7]	2 (3.8%) [0.5–13.0]	1 (2.6%) [0.1–13.5]	0 [0.0–18.5]	1 (3.1%) [0.1–16.2]
Progression-free survival						
Median	14.3 months [11.2–17.5]	11.8 months [8.3–14.9]	16.8 months [11.0–20.5]	13.6 months [8.3–20.3]	14.3 months [10.5–17.5]	15.5 months [10.4–23.8]
Number of patients with events	74 (69.8%)	36 (81.8%)	38 (61.3%)	32 (71.1%)	18 (72.0%)	24 (66.7%)
Number of patients censored	32 (30.2%)	8 (18.2%)	24 (38.7%)	13 (28.9%)	7 (28.0%)	12 (33.3%)

Data are reported number (%) [95% CI] for best overall response and median number of months [95% CI] or number (%) for progression-free survival. Best overall response was assessed only in patients of the intent-to-treat population with measurable disease at baseline. Progression-free survival was assessed in the intent-to-treat population. One patient had a missing progesterone receptor score and was considered as having a negative score

### Exploratory and sensitivity analyses

Predefined exploratory subgroup analyses for ORR and PFS stratified by prior trastuzumab treatment and by hormone receptor status are shown in Table 2.

A sensitivity analysis excluding patients with tumor assessments performed after the intake of new anticancer therapy broadly supported the primary analysis for ORR, median PFS, and median TTP (Table 3). A second sensitivity analysis, including progressive disease due to symptomatic deterioration, was also consistent with the primary PFS and TTP analyses (Table 3).

**Table 3:** Sensitivity analyses of best overall response, progression-free survival, and time to progression, intent-to-treat population

	Cohort 1: pertuzumab, trastuzumab, and vinorelbine	
	Sensitivity analyses	
	Excluding tumor assessments after intake of any new anticancer therapy N = 106	Including progressive disease due to symptomatic deterioration N = 106
Best overall response		ND^a^
Patients with measurable disease at baseline	89 (84.0%)	
Overall response rate	57 (64.0%) [53.2–73.9]	
Complete response	10 (11.2%) [5.5–19.7]	
Partial response	47 (52.8%) [41.9–63.5]	
Stable disease	17 (19.1%) [11.5–28.8]	
Progressive disease	5 (5.6%) [1.8–12.6]	
Not evaluable	10 (11.2%) [5.5–19.7]	
Progression-free survival		
Median	12.5 months [10.4–16.8]	13.8 months [11.0–17.3]
Number of patients with events	65 (61.3%)	74 (69.8%)
Number of patients censored	41 (38.7%)	32 (30.2%)
Time to progression		
Median	12.9 months [10.5–16.8]	14.3 months [11.2–17.5]
Number of patients with events	62 (58.5%)	72 (67.9%)
Number of patients censored	44 (41.5%)	34 (32.1%)

Data are reported number (%) [95% CI] for best overall response and median number of months [95% CI] or number (%) for progression-free survival and time to progression. Best overall response was assessed only in patients of the intent-to-treat population with measurable disease at baseline. Progression-free survival and time to progression were assessed in the intent-to-treat population

^a^A sensitivity analysis including progressive disease due to symptomatic deterioration was not performed for best overall response

### Safety

Overall, study treatment was reasonably well tolerated, with no unexpected toxicities. Table 4 lists AEs of any grade with an incidence of ≥20%; almost all patients experienced an AE (105/106 patients, 99.1%). Diarrhea (61/106 patients, 57.5%) and neutropenia (54/106 patients, 50.9%) were the most common AEs. Grade 3 or higher AEs (Table 5) were reported in 64/106 (60.4%) patients; neutropenia (33/106 patients, 31.1%) and leukopenia (14/106 patients, 13.2%) occurred most frequently. Granulocyte colony-stimulating factors (G-CSFs) were administered concomitantly in 28/106 (26.4%) patients for the management of neutropenia. Thirty-two of 106 (30.2%) patients experienced at least one SAE (Table 5), with febrile neutropenia and hypersensitivity being the only SAEs experienced by more than two patients. Investigator-assessed pertuzumab-related AEs occurred in 70/106 (66.0%) patients, trastuzumab-related AEs in 79/106 (74.5%) patients, and vinorelbine-related AEs in 96/106 (90.6%) patients. AEs led to study drug interruption in 77/106 (72.6%) patients and discontinuation in 36/106 (34.0%) patients: discontinuation of pertuzumab, trastuzumab, and vinorelbine in 18/106 (17.0%), 16/106 (15.1%), and 33/106 (31.1%) patients, respectively. Neutropenia led to vinorelbine discontinuation in 4/106 (3.8%) patients; no patients discontinued treatment due to febrile neutropenia, leukopenia, or diarrhea. Of the 23 deaths that occurred in the safety population, 18/23 (78.3%) were from disease progression and there was one case each of myocardial infarction (4.3%), septic shock (4.3%), and pneumonia (4.3%). For the remaining two patients (8.7%) the cause of death was unknown.

**Table 4:** Adverse events (any grade) with an incidence of ≥20%, safety population

Adverse event	Cohort 1: pertuzumab, trastuzumab, and vinorelbine N = 106
Any adverse event	105 (99.1%)
Diarrhea	61 (57.5%)
Neutropenia	54 (50.9%)
Nausea	52 (49.1%)
Asthenia	42 (39.6%)
Pyrexia	37 (34.9%)
Anemia	36 (34.0%)
Fatigue	36 (34.0%)
Constipation	35 (33.0%)
Vomiting	34 (32.1%)
Chills	30 (28.3%)
Alopecia	27 (25.5%)
Rash	25 (23.6%)
Leukopenia	24 (22.6%)
Decreased appetite	23 (21.7%)
Weight decreased	22 (20.8%)

Data are reported number (%)

**Table 5:** Adverse events of grade ≥3 with an incidence of ≥3% and serious adverse events in >1 patient, safety population

Adverse event	Cohort 1: pertuzumab, trastuzumab, and vinorelbine N = 106
Any grade ≥3 adverse event	64 (60.4%)
Neutropenia	33 (31.1%)
Leukopenia	14 (13 · 2%)
Diarrhea	7 (6.6%)
Anemia	6 (5.7%)
Febrile neutropenia	6 (5.7%)
Asthenia	5 (4.7%)
Constipation	4 (3.8%)
Fatigue	4 (3.8%)
Total number of serious adverse events	44
Number of patients with ≥1 serious adverse event	32 (30.2%)
Febrile neutropenia	6 (5.7%)
Hypersensitivity	5 (4.7%)
Abdominal pain	2 (1.9%)
Drug hypersensitivity	2 (1.9%)
Pneumonia	2 (1.9%)
Pyrexia	2 (1.9%)
Septic shock	2 (1.9%)^a^
Adverse events leading to death (grade 5)	2 (1.9%)^a–c^

Data reported are n (%)

^a^One patient died from septic shock while on treatment

^b^One patient died of a myocardial infarction while on treatment

^c^A further 21 deaths were recorded at the end of the study but they did not occur while patients were on study treatment; one death due to pneumonia, two deaths due to unknown causes, and 18 deaths due to disease progression

Cardiac disorders occurred in 16/106 (15.1%) patients. In three patients these were grade ≥3: grade 3 left ventricular dysfunction in one (0.9%) patient, grade 3 supraventricular tachycardia in one (0.9%) patient and grade 5 myocardial infarction in one (0.9%) patient. Eighteen of 106 (17.0%) patients had AEs suggestive of CHF: nine (8.5%) patients had LVEF declines (of which four [3.8%] had declines to <45%), two (1.9%) patients had left ventricular dysfunction, six (5.7%) patients had peripheral edema, one (0.9%) patient had orthopnea, and three (2.8%) patients had peripheral swelling (one patient had three events [LVEF decline, peripheral edema, and orthopnea] and one patient had two events [LVEF decline and peripheral edema]). However, there were no confirmed cases of CHF. Aside from the nine patients above, a further two (1.9%) experienced LVEF declines to <45%; all but two patients’ LVEF recovered to ≥50%.

### Anticancer therapies after discontinuation of study treatment

Ninety of 106 (84.9%) patients received anticancer therapies after discontinuing study treatment (Table 6). Of these 90 patients, 57 (63.3%) received trastuzumab, 30 (33.3%) capecitabine, 27 (30.0%) lapatinib, 24 (26.7%) taxanes, and 17 (18.9%) trastuzumab emtansine.

**Table 6:** Anticancer treatments received by patients after discontinuing study treatment, safety population

INN class/preferred term^a^	Cohort 1: pertuzumab, trastuzumab, and vinorelbine N = 106^b^ n = 90^c^
HER2-targeted treatment^d,e^ (also counted in individual classes)	
Patients who received any treatment	81 (90.0%)
Lapatinib	27 (30.0%)
Neratinib	1 (1.1%)
Pertuzumab	10 (11.1%)
Trastuzumab	57 (63.3%)
Trastuzumab emtansine	17 (18.9%)
Alkylating agents	
Patients who received any treatment	6 (6.7%)
Cyclophosphamide	6 (6.7%)
Antiestrogens	
Patients who received any treatment	6 (6.7%)
Fulvestrant	2 (2.2%)
Tamoxifen	4 (4.4%)
Antimetabolites	
Patients who received any treatment	32 (35.6%)
Capecitabine	30 (33.3%)
Fluorouracil	1 (1.1%)
Gemcitabine	3 (3.3%)
Antineoplastic agents^f^	
Patients who received any treatment	19 (21.1%)
Eribulin	3 (3.3%)
Temsirolimus	1 (1.1%)
Trastuzumab emtansine	17 (18.9%)
Aromatase inhibitors	
Patients who received any treatment	15 (16.7%)
Anastrozole	5 (5.6%)
Exemestane	5 (5.6%)
Letrozole	5 (5.6%)
Cytotoxic antibiotics	
Patients who received any treatment	9 (10.0%)
Doxorubicin	4 (4.4%)
Epirubicin	4 (4.4%)
Mitoxantrone	1 (1.1%)
Gonadotrophin and analogs	
Patients who received any treatment	2 (2.2%)
Leuprorelin	2 (2.2%)
Monoclonal antibodies	
Patients who received any treatment	57 (63.3%)
Denosumab	2 (2.2%)
Pertuzumab	10 (11.1%)
Trastuzumab	57 (63.3%)
Penicillins	
Patients who received any treatment	1 (1.1%)
Dicloxacillin	1 (1.1%)
Platinum compounds	
Patients who received any treatment	5 (5.6%)
Carboplatin	3 (3.3%)
Cisplatin	2 (2.2%)
Surgical and medical procedures	
Patients who received any treatment	22 (24.4%)
Brain tumor operation	1 (1.1%)
Breast operation	1 (1.1%)
Gamma radiation therapy to brain	1 (1.1%)
Lesion excision	1 (1.1%)
Lymphadenectomy	1 (1.1%)
Malignant tumor excision	2 (2.2%)
Mastectomy	5 (5.6%)
Radiotherapy	8 (8.9%)
Radiotherapy to brain	5 (5.6%)
Radiotherapy to lung	1 (1.1%)
Radiotherapy to lymph nodes	1 (1.1%)
Taxanes	
Patients who received any treatment	24 (26.7%)
Docetaxel	10 (11.1%)
Paclitaxel	15 (16.7%)
Topoisomerase inhibitors	
Patients who received any treatment	1 (1.1%)
Etoposide	1 (1.1%)
Tyrosine kinase inhibitors	
Patients who received any treatment	28 (31.1%)
Lapatinib	27 (30.0%)
Neratinib	1 (1.1%)
Vinca alkaloids	
Patients who received any treatment	11 (12.2%)
Vinorelbine^g^	11 (12.2%)
Combinations^d^ (also counted in individual classes)	
Patients who received any treatment	5 (5.6%)
Cisplatin/cyclophosphamide/epirubicin/etoposide/fluorouracil	1 (1.1%)
Dicloxacillin/doxorubicin	1 (1.1%)
Capecitabine/lapatinib/trastuzumab	1 (1.1%)
Carboplatin/gemcitabine	1 (1.1%)
Carboplatin/trastuzumab	1 (1.1%)

Data reported are number (%).

^a^INN classes are presented alphabetically and preferred terms are sorted within INN classes alphabetically

^b^Number of patients in the safety population

^c^Number of patients who received at least one anticancer treatment after discontinuing study treatment. Percentages are based on n. Patients may have received more than one anticancer treatment after discontinuing study treatment. Some therapies began before the last study treatment was received

^d^All preferred terms within the “HER2-targeted treatment” and “Combinations” summaries are also included in their respective INN classes

^e^The “HER2-targeted treatment” summary is a reclassification of specific preferred terms, identified by the medical team

^f^The “Antineoplastic agents” summary includes any drug used to treat cancers that cannot be assigned to a more specific pharmacological class

^g^Eleven patients had vinorelbine (re)introduced after study treatment was discontinued: six patients who had stopped study treatment due to an adverse event/unacceptable toxicity (three of whom had not received on study vinorelbine due to AE with pertuzumab and/or trastuzumab administration and had discontinued study treatment before receiving vinorelbine as an anticancer therapy); three patients who had stopped study treatment due to administrative/other reasons; and two patients who had stopped study treatment due to disease progression

## Discussion

The combination of pertuzumab, trastuzumab, and docetaxel is standard of care for first-line treatment of HER2-positive metastatic breast cancer [24]. Although docetaxel is an active and well-established chemotherapy it may not always be patients’ or physicians’ preference due to its toxicity profile or due to prior docetaxel treatment. Therefore, alternative chemotherapy partners, such as vinorelbine, are needed to increase options and convenience.

VELVET is the first clinical trial to assess a non-taxane-based chemotherapy in combination with pertuzumab and trastuzumab for the first-line treatment of HER2-positive locally advanced or metastatic breast cancer. As VELVET was a single-arm proof-of-concept study, ORR, defined by RECIST guidelines, was considered to be an appropriate endpoint to directly measure the study treatment effect in a timely manner. Here we report the results from Cohort 1 only, where the study drugs were given sequentially as separate infusions as per current clinical practice.

The combination of pertuzumab, trastuzumab, and vinorelbine resulted in an investigator-assessed ORR of 74.2% (95% CI 63.8–82.9, 13.5% complete response and 60.7% partial response) and a median time to response of 2.1 months (range 0.0–29.0) in patients with measurable disease at baseline. The median PFS was 14.3 months (95% CI 11.2–17.5), the median TTP was 14.9 months (95% CI 11.3–17.9), and the median DoR in responders was 13.3 months (range 2.1–29.5).

The results of VELVET Cohort 1 show that the combination of vinorelbine plus trastuzumab and pertuzumab is active in the first-line setting and that the addition of pertuzumab does not markedly change the toxicity profile of vinorelbine plus trastuzumab. Efficacy appeared not to differ greatly versus the results of a prior study with first-line trastuzumab and vinorelbine (HERNATA), which demonstrated that this combination was an active and well-tolerated alternative to trastuzumab and docetaxel for first-line treatment of HER2-positive locally advanced or metastatic breast cancer [22]. However, as VELVET had no control arm, it is not possible to know what the activity of vinorelbine and trastuzumab without pertuzumab would be in the VELVET population.

The efficacy reported in VELVET also appears lower than that observed in CLEOPATRA where docetaxel was used as the chemotherapy partner for pertuzumab and trastuzumab; while ORR was broadly similar, median PFS and DoR looked shorter when vinorelbine was combined with the two monoclonal antibodies rather than docetaxel [7, 8]. However, caution must be exercised when doing cross-trial comparisons, given the significantly different study designs (randomized phase III versus single-arm phase II) and primary endpoints (PFS in CLEOPATRA and TTP in HERNATA versus ORR in VELVET), different patient populations (VELVET had no Asian patients, and had a higher number of hormone receptor-positive patients and a higher frequency of patients with visceral disease at baseline than CLEOPATRA), and the different number and type of prior anticancer therapies [22].

Importantly, a high percentage of patients in VELVET Cohort 1 (41.5%) received prior neoadjuvant or adjuvant trastuzumab, which makes VELVET more representative of current real-world patient populations than earlier trials. However, unexpectedly, based on current understanding of the potential mechanisms of resistance to HER2-directed therapies [25], the ORR in VELVET Cohort 1 was slightly higher in patients who had prior trastuzumab treatment than in trastuzumab-naïve patients. Overall, given the exploratory nature of this subgroup analysis, the small numbers of patients, and wide overlapping CIs, this finding should be interpreted with caution.

Sensitivity analyses (excluding tumor assessments performed after the intake of new anticancer therapy, and including progressive disease due to symptomatic deterioration) to test the robustness of ORR, PFS, and TTP were broadly consistent with the primary efficacy analyses. Two-year survival was 78.3% at clinical cutoff. Median OS, a secondary endpoint, had not been reached, suggesting 2 years is too short for OS follow-up.

The treatment regimen was reasonably well tolerated with no unexpected toxicities, and consistent with the known safety profiles of the individual agents, and with the favorable toxicity observed in clinical trials combining trastuzumab and vinorelbine versus other chemotherapy options in the HER2-positive metastatic setting [12–22]. Diarrhea and neutropenia were the most common AEs. The incidence of grade ≥3 diarrhea was low and no patients discontinued treatment from it. The incidence of grade ≥3 neutropenia was 31.1%, but this was managed with G-CSFs and only four (3.8%) patients discontinued treatment due to neutropenia. While acknowledging the caveats of cross-trial comparisons, as expected the incidence of grade ≥3 neutropenia and febrile neutropenia was lower with pertuzumab, trastuzumab, and vinorelbine than that reported for pertuzumab, trastuzumab, and docetaxel in CLEOPATRA (31.1% versus 48.9%, and 5.7% versus 13.8%, respectively). The incidence of grade ≥3 diarrhea was similar (6.6% versus 7.9%), and the incidence of grade ≥3 asthenia slightly higher in VELVET Cohort 1 (4.7% versus 2.5%) [7]. The safety findings are not surprising given the well-documented toxicity profiles of docetaxel [9] and vinorelbine [26].

The relatively small number of patients and the lack of a comparator arm may limit the interpretation of this study. However, a single-arm phase II study such as VELVET may be sufficient to provide insights on the efficacy and safety of a novel regimen, particularly as large clinical trial datasets of the investigational drugs are already available in similar therapeutic settings.

Beyond VELVET, one small phase II study has investigated paclitaxel as an alternative to docetaxel for use in combination with pertuzumab and trastuzumab for the treatment of HER2-positive metastatic breast cancer [27]. Results showed that this combination was highly active and well tolerated, with a low incidence of AEs [27]. Additionally, preliminary safety results from the ongoing phase IIIb PERUSE study (NCT01572038) have shown that both paclitaxel and nab-paclitaxel appear to be feasible and tolerable chemotherapy partners for trastuzumab and pertuzumab for first-line HER2-positive advanced breast cancer, with no unexpected safety signals observed [28].

## Conclusions

In summary, the combination of pertuzumab, trastuzumab, and vinorelbine, given as separate infusions, appears to be active and reasonably well tolerated for first-line treatment of HER2-positive locally advanced or metastatic breast cancer, and offers a viable alternative to the standard of care (pertuzumab, trastuzumab, and docetaxel) for patients who cannot receive docetaxel.

## Additional file

Additional file 1: VELVET: list of study investigators and central ethics committees. (PDF 21 kb)

## Abbreviations

AEs: adverse events
BOR: best overall response
CHF: congestive heart failure
CI: confidence interval
DoR: duration of response
ECOG: Eastern Cooperative Oncology Group
HRQoL: health-related quality of life
IDMC: independent data monitoring committee
ITT: intent-to-treat
LVEF: left ventricular ejection fraction
MBC: metastatic breast cancer
MedDRA: Medical Dictionary for Regulatory Activities
ORR: objective response rate
OS: overall survival
PFS: progression-free survival
RECIST: Response Evaluation Criteria In Solid Tumors
SAEs: serious adverse events
TTP: time to progression
